# Combination of Chinese Herbal Medicines and Conventional Treatment versus Conventional Treatment Alone in Patients with Acute Coronary Syndrome after Percutaneous Coronary Intervention (5C Trial): An Open-Label Randomized Controlled, Multicenter Study

**DOI:** 10.1155/2013/741518

**Published:** 2013-07-02

**Authors:** Shao-Li Wang, Cheng-Long Wang, Pei-Li Wang, Hao Xu, Hong-Ying Liu, Jian-Peng Du, Da-Wu Zhang, Zhu-Ye Gao, Lei Zhang, Chang-Geng Fu, Shu-Zheng Lü, Shi-Jie You, Jun-Bo Ge, Tian-Chang Li, Xian Wang, Guan-Lin Yang, Hong-Xu Liu, Jing-Yuan Mao, Rui-Jie Li, Li-Dian Chen, Shu Lu, Da-Zhuo Shi, Ke-Ji Chen

**Affiliations:** ^1^Guang'anmen Hospital, China Academy of Chinese Medical Sciences, Beijing 100053, China; ^2^Xiyuan Hospital, China Academy of Chinese Medical Sciences, Beijing 100091, China; ^3^Zhongshan Hospital, Fudan University, Shanghai 200032, China; ^4^Beijing Anzhen Hospital, Beijing Institute of Respiratory Medicine, Capital Medical University, Beijing 10029, China; ^5^Cardiovascular Institute & Fuwai Hospital, Chinese Academy of Medical Sciences & Peking Union Medical College, Beijing 100037, China; ^6^Beijing Tongren Hospital, Capital Medical University, Beijing 100730, China; ^7^Dongzhimen Hospital, Beijing University of Chinese Medicine, Beijing 100007, China; ^8^The Affiliated Hospital of Liaoning Traditional Chinese Medicine University, Shenyang 110033, China; ^9^Beijing Chinese Medicine Hospital, Capital Medical University, Beijing 100010, China; ^10^First Teaching Hospital of Tianjin University of Traditional Chinese Medicine, Tianjin 300193, China; ^11^Beijing Chuiyangliu Hospital, Beijing 100022, China; ^12^The Second People's Hospital of Fujian Province, Fuzhou 350100, China; ^13^Wuxi Traditional Chinese Medicine Hospital, Nanjing University of Traditional Chinese Medicine, Wuxi 214001, China

## Abstract

*Aims.* To evaluate the efficacy of Chinese herbal medicines (CHMs) plus conventional treatment in patients with acute coronary syndrome (ACS) after percutaneous coronary intervention (PCI). *Methods and Results.* Participants (*n* = 808) with ACS who underwent PCI from thirteen hospitals of mainland China were randomized into two groups: CHMs plus conventional treatment group (treatment group) or conventional treatment alone group (control group). All participants received conventional treatment, and participants in treatment group additionally received CHMs for six months. The primary endpoint was the composite of cardiac death, nonfatal recurrent MI, and ischemia-driven revascularization. Secondary endpoint was the composite of readmission for ACS, stroke, or congestive heart failure. The safety endpoint involved occurrence of major bleeding events. The incidence of primary endpoint was 2.7% in treatment group versus 6.2% in control group (HR, 0.43; 95% CI, 0.21 to 0.87; *P* = 0.015). The incidence of secondary endpoint was 3.5% in treatment group versus 8.7% in control group (HR, 0.39; 95% CI, 0.21 to 0.72; *P* = 0.002). No major bleeding events were observed in any participant. *Conclusion.* Treatment with CHMs plus conventional treatment further reduced the occurrence of cardiovascular events in patients with ACS after PCI without increasing risk of major bleeding.

## 1. Introduction

Acute coronary syndrome (ACS), encompassing unstable angina (UA) and acute myocardial infarction (AMI, non-ST elevation, and ST elevation), is one of the leading causes of morbidity and mortality. There has been a steady decline in mortality from coronary artery disease (CAD) in most developed countries over the last three decades [1], primarily due to dramatic advances in revascularization procedures such as percutaneous coronary intervention (PCI) and coronary artery bypass graft (CABG), as well as pharmacological treatments [2]. Approximately 10% of ACS survivors after PCI, however, will ultimately suffer a second AMI, stroke, or cardiovascular death [3, 4] despite the availability of timely, appropriate treatments. Therefore, reducing the risk of recurrent cardiovascular events in patients with ACS after PCI remains a great challenge in the foreseeable future [5].

Chinese herbal medicines (CHMs) have been widely used in clinical practice for thousands of years. In the past few decades, CHMs have shown beneficial effects in improving clinical symptoms and clinical outcomes in CAD patients. Xinyue Capsule and Fufang *Chuanxiong* Capsule are commonly prescribed in mainland China, and both have been approved by the State Food and Drug Administration (SFDA) of China for clinical use in CAD patients. The major therapeutic effects of these drugs, as documented in previous trials, include relieving myocardial ischemia, decreasing symptoms, improving myocardial reperfusion after PCI, regulating blood lipids, and reducing recurrent angina [6–9]. However, no study has yet to focus on the efficacy of the two capsules in reducing recurrence of cardiovascular events in patients with ACS after PCI. Therefore, in this multicenter, open-label, randomized controlled trial (chictr.org number: ChiCTR-TRC-00000021), we evaluated the efficacy of Xinyue Capsule and Fufang *Chuanxiong* Capsule plus conventional treatment on cardiovascular events in patients with ACS after PCI. 

## 2. Method

### 2.1. Design Overview

This study was conducted at thirteen hospitals in five provinces of mainland China. The participants were recruited from April 2008 to October 2009, and follow-up was completed by October 2010. The study protocol was approved by the ethics review board of Xiyuan Hospital, China Academy of Chinese Medical Sciences (CACMS), in accordance with the principles described in the *Declaration of Helsinki* [10], and all participants signed informed consent forms before enrollment.

### 2.2. Setting and Participants

Recruitment, intervention, and data collection were performed at the thirteen participating hospitals. Patients between 18 and 75 years of age were eligible for inclusion if they were hospitalized for ACS [11, 12] involving either AMI (with or without ST segment elevation) or UA and also underwent successful PCI (defined as the target vessel with TIMI grade 3 flow). The exclusion criteria were as follows: (1) concomitant affliction with severe complications including hepatic, renal, and hematopoietic dysfunction, psychiatric disorders, or cancers; (2) absence of written informed consent, unwillingness to participate in follow-up, or refusal to receive treatment with study drugs; (3) pregnancy or breastfeeding; and (4) concurrent enrollment in other clinical studies.

### 2.3. Randomization and Intervention

An independent, off-site clinical trials statistician at CACMS used a computer-generated random allocation sequence to randomize the trial in blocks of four, stratified with each recruiting center. The details of the sequence remained unknown to any investigator or coordinator and were contained in sequentially numbered, opaque, sealed envelopes (SNOSE), bearing only the hospital name and a number on the outside. A pharmacist at each center who was independent of the clinical study kept the allocation sequence, took responsibility for the allocation, and prepared the treatment medication. After completing the baseline visit, participants who met the enrollment criteria were randomly assigned in a 1 : 1 ratio to receive either CHMs (Xinyue Capsule and Fufang *Chuanxiong* Capsule) plus conventional treatment or conventional treatment alone. Participants and investigators were masked to the treatment allocation until interventions were assigned. Data collectors and outcome adjudicators were masked until all data were entered into the database. Data management and statistical analyses were performed solely by data handlers and data analysts at Beijing Jiaotong University who were masked to the treatment assignments until the statistical report was completed. The study was open-label because the unique aroma and taste of Fufang *Chuanxiong* Capsule and Xinyue Capsule significantly challenged the successful blinding. In addition, even if we designed placebo capsules for the present study, the participants could easily distinguish between placebo and true capsules by the specific aftertaste left from oral intake of the true capsules.

All participants received conventional treatment in accordance with current guidelines [11, 12], including aspirin (100 mg/day indefinitely), clopidogrel (75 mg/day for at least 12 months), and statins. All other medications were decided by physicians at each center who were not involved in the study. After participants were discharged, medication decisions and the option of revascularization were made by the responsible clinician without restriction. Angiographic follow-up was performed during the follow-up period, but it was not required in this study.

In addition to the conventional treatment, participants in the treatment group received Xinyue Capsule (two capsules orally, three times daily) and Fufang *Chuanxiong* Capsule (two capsules orally, three times daily) for six successive months. The Xinyue Capsule (SFDA Registry number: Z20030073; manufacturer: Jilin Jian Yisheng Pharmaceutical Co., Ltd., Jian City, Jilin Province, China) is an extract from leaves and stems of *Panax quinquefolius* L., containing 50 mg total ginsenosides. The Fufang *Chuanxiong* Capsule (SFDA Registry number: 0802205; manufacturer: Shandong Phoenix Pharmaceutical Co., Ltd., Dongying City, Shandong Province, China) is made from *Chuanxiong* and *Ligusticum*, with each capsule containing 3.20 mg/gm ligustrazine and 1.73 mg/gm ferulic acid. The quality of the two CHMs met the Chinese Medicine Standards of the SFDA. Capsules were distributed to the thirteen study sites with the same batch number. The companies that provided the two CHMs had no role in the design, analysis, or interpretation of the study. 

From the baseline visit to the end of the study, the other CHMs used in the treatment of ACS after PCI, which might complicate the pharmacological effectiveness of Xinyue Capsule or Fufang *Chuanxiong* Capsule, were prohibited. A member of the executive committee in the study was responsible for monitoring quality control with respect to the management of all participants. The adherence of participants to study medication was assessed by independent nurses at each site.

### 2.4. Outcomes and Follow-Up

At the baseline visit, investigators assessed the following characteristics which might have an impact on treatment: body mass index (BMI), heart rate, the number of diseased vessels, target vessels, smoking history, presence or absence of diabetes, hypertension, hyperlipidemia, CAD family history and medications, and so forth. The primary and secondary endpoints were adjudicated at 30 days, as well as at 3, 6, 9, and 12 months after the baseline visit.

The primary endpoint was the composite of cardiac death, nonfatal recurrent MI, or ischemia-driven revascularization. The secondary endpoint was the composite of readmission for ACS, stroke, or congestive HF. The safety endpoint concerned major bleeding events, defined as any intracranial bleeding, or any clinically relevant bleeding necessitated a blood transfusion judged by the investigators. All deaths were considered cardiac unless an unequivocal noncardiac cause was identified. Ischemia-driven revascularization was defined as repeat revascularization with either PCI or CABG because of recurrent myocardial ischemic events. Repeat PCI was defined as revascularization of target lesions or target vessels. Stroke was defined as the development of disabling neurologic symptoms with objective findings lasting at least 24 hours. Recurrent MI was diagnosed based on reappearance of symptoms, and/or new electrocardiographic changes in association with a reelevation of creatine kinase-MB (CK-MB) to levels greater than three times the upper limit of the reference level. Congestive HF was defined as a new diagnosis of congestive HF requiring hospitalization. 

Subjects were followed up at each study center. The endpoint data were collected and recorded in a case report form (CRF) by the investigators at each visit (either a direct visit or telephone interview). For remote participants interviewed by telephone, local medical reports were collected by mail and a direct visit was performed at least once during the one-year follow-up period. All clinical outcomes were adjudicated by independent outcome committees whose members were blinded to treatment assignment with review of original documentation.

### 2.5. Statistical Analysis

Sample size calculations were based on evidence from previous studies, which showed that the one-year composite incidence of cardiac death, nonfatal recurrent MI, or ischemia-driven revascularization in patients with ACS after PCI treated by conventional treatment was 8% to 18% [13, 14]. Thus the incidence of the primary endpoint in this study during one-year in the control group was estimated at 13.5%, and treatment with additional CHMs reduced it to 7% [15]. For our study, a total of 676 participants would provide 80% power to test a difference in the primary endpoint at the 5%, two-sided level of significance. Allowing for a 20% dropout rate and adding power for analysis of the secondary endpoint, we recruited 808 total participants. 

All participants were subject to baseline analysis as well as efficacy and safety evaluations. All data analysis was conducted according to a preestablished analysis plan. For categorical variables, the data were presented in a frequency table and expressed as percentages, and intergroup differences were compared by Chi-square or Fisher exact tests. For continuous variables, mean and standard deviation was used for normally distributed data, and median with interquartile range was calculated for not normally distributed data. A Student's *t*-test or Wilcoxon Rank-sum test was used, as appropriate, for the analyses of intergroup differences. The difference in cumulative incidence of the primary or secondary endpoints at one-year between groups was estimated by the Kaplan-Meier method with the log-rank test. The treatment efficacy, as measured by the hazard ratio (HR) and its associated 95% confidence interval (CI), was estimated with the Cox proportional hazards regression. For the calculation of an adjusted HR with 95% CI for the primary or secondary endpoints, Cox proportional hazards regression was performed with 11 preidentified covariates of interest: age, gender, number of diseased vessels, target vessels, final diagnosis, smoking history, CAD family history, BMI, number of randomization centers, presence or absence of diabetes, hypertension, and hyperlipidemia. Participants who were lost to follow-up were censored at their last visit. The intention-to-treat method was applied in the analysis.

A two-sided *P* value less than 0.05 was considered to be statistically significant. The statistical analysis was performed with SPSS statistical software, Version 17.0 for Windows.

## 3. Results

### 3.1. Participant Characteristics

**Figure 1:**
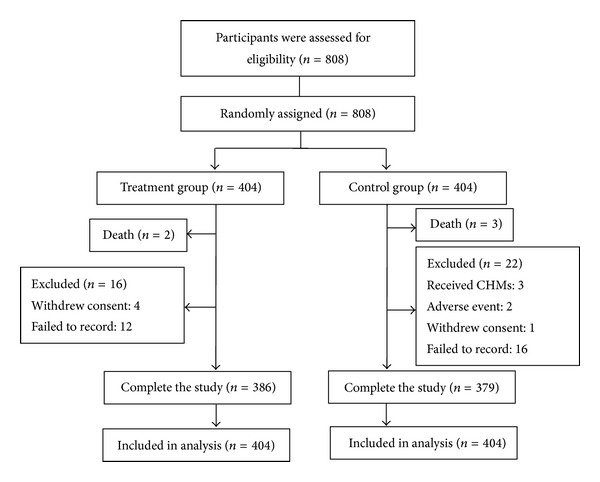
Study flow diagram.

In this study, 808 participants with ACS after successful PCI from April 2008 to October 2010 were assigned to the control (404 participants) and treatment (404 participants) groups randomly. During follow-up, three participants died of cardiac events (two in the treatment group and one in the control group) and two participants in the control group died of cancer (both due to lung cancer). Thirty eight participants (4.7%) were classified as dropout with no significant difference between the two groups [16 (4.0%) in the treatment group versus 22 (5.4%) in the control group, *P* = 0.319]. Among the dropouts, five declined to participate in the follow-up, two had noncardiac adverse events (cancer), twenty-eight were unreachable for data collection, and three in the control group received CHMs were excluded. A total of 765 participants completed the one-year follow-up (Figure 1). CHMs were administered to 378 (93.6%) participants in the treatment group for the six months. The baseline characteristics of the participants are shown in Table 1, and the two groups were well matched, except for the proportion of male participants.

### 3.2. Primary Endpoint

**Figure 2:**
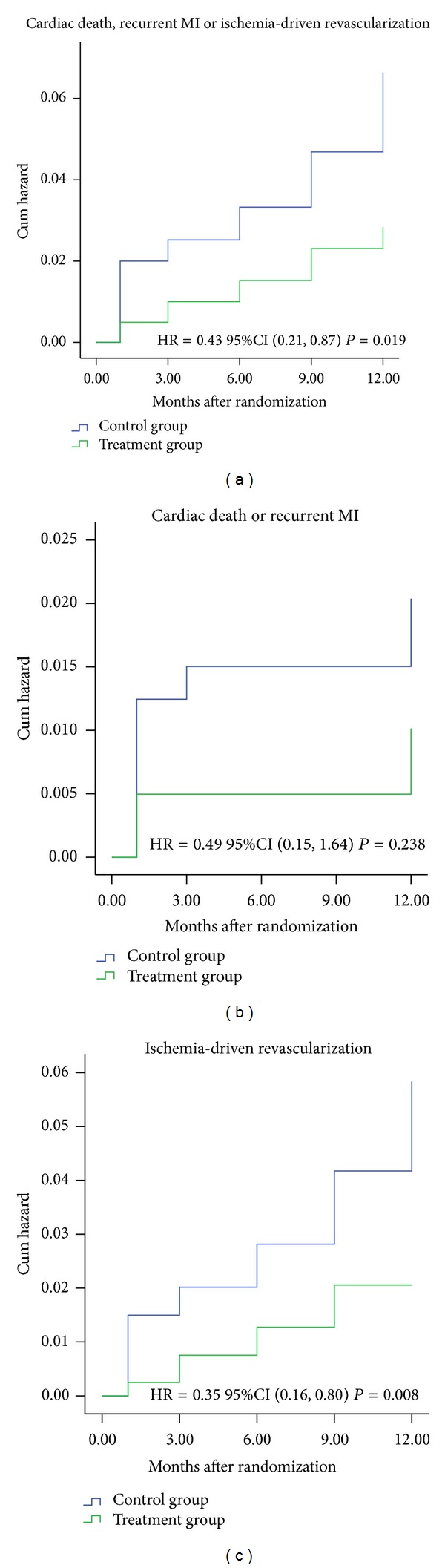
Kaplan-Meier time to event curve for primary endpoint.

During the follow-up period, the cumulative incidence of the primary endpoint in the treatment group was significantly lower than that in the control group [11 (2.7%) versus 25 (6.2%); unadjusted HR 0.43, 95% CI 0.21 to 0.87, *P* = 0.015]. After adjusting for the effects of covariates, the combination of CHMs with conventional treatment was associated with a significant reduction in the primary endpoint compared to conventional treatment alone (adjusted HR 0.44, 95% CI 0.21 to 0.92, *P* = 0.028) (Table 2 and Figure 2(a)). Among the components of the primary endpoint, cardiac death and recurrent MI did not differ significantly between the treatment and control groups (1.0% versus 2.0%, unadjusted HR 0.49, 95% CI 0.15 to 1.64, adjusted HR 0.35, 95% CI 0.09 to 1.33, *P* = 0.238) (Table 2 and Figure 2(b)). Ischemia-driven revascularization, however, was significantly reduced in the treatment group compared to the control group (2.0% versus 5.4%, unadjusted HR 0.35, 95% CI 0.16 to 0.80, adjusted HR 0.36, 95% CI 0.16 to 0.82, *P* = 0.008) (Table 2 and Figure 2(c)).

### 3.3. Secondary Endpoint

**Figure 3:**
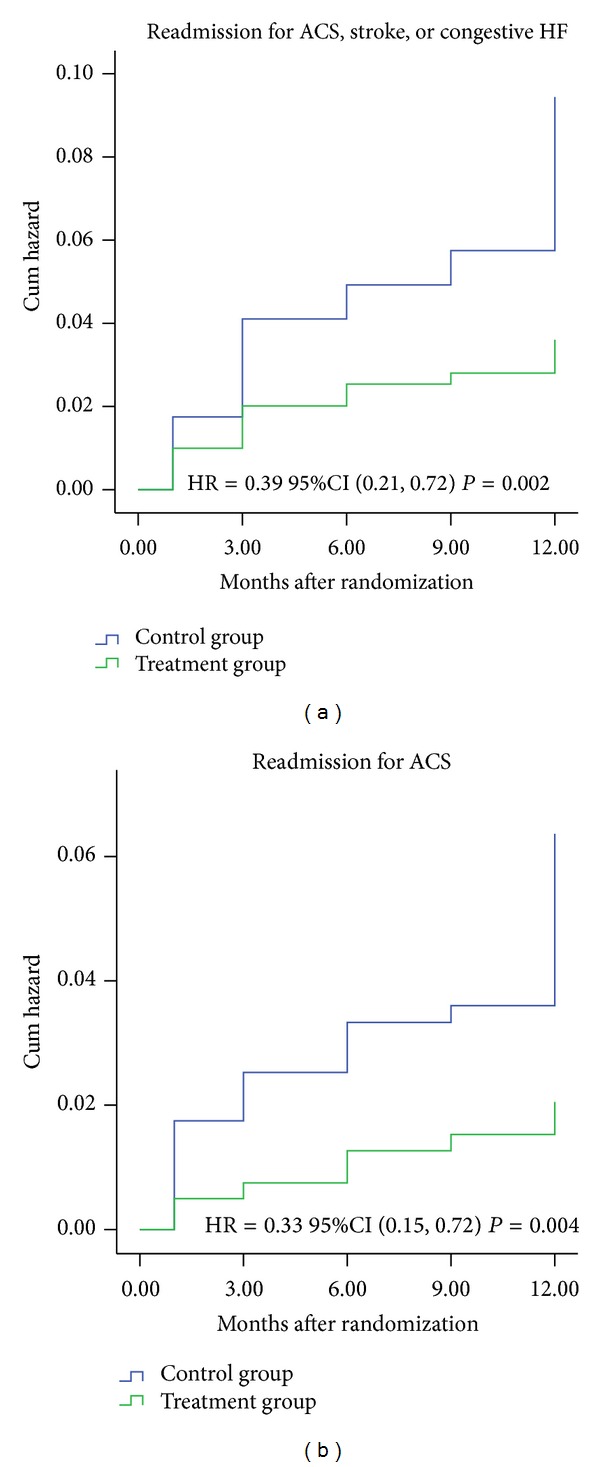
Kaplan-Meier time to event curve for secondary endpoint.

The secondary endpoints occurred in 14 (3.5%) in the treatment group and 35 (8.7%) in the control group (unadjusted HR 0.39, 95% CI 0.21 to 0.72, *P* = 0.002). After adjusting for the effects of covariates, the addition of CHMs to conventional treatment was associated with a significant reduction in the secondary endpoint compared with conventional treatment alone (adjusted HR 0.37, 95% CI 0.21 to 0.72, *P* = 0.002) (Table 2 and Figure 3(a)). Among the components of the endpoint, the cumulative incidence of readmission for ACS in the treatment group was lower than that in the control group (2.0% versus 5.9%, unadjusted HR 0.33, 95% CI 0.15 to 0.72, adjusted HR 0.29, 95% CI 0.13 to 0.65, *P* = 0.004) (Table 2 and Figure 3(b)). However, the incidence of stroke (0.7% versus 1.5%, unadjusted HR 0.49, 95% CI 0.12 to 0.97, adjusted HR 0.69, 95% CI 0.16 to 3.02, *P* = 0.307) and congestive HF (0.7% versus 1.2%, unadjusted HR 0.59, 95% CI 0.14 to 2.48, adjusted HR 0.52, 95% CI 0.12 to 2.36, *P* = 0.469) did not differ between the two groups.

### 3.4. Safety

Major bleeding events were not observed in all participants. Aside from the cardiovascular events defined as primary and secondary endpoints in this study, four participants in the control group were afflicted with cancer. One dropped out due to esophageal cancer, one due to thyroid cancer, and two died of lung cancer. In the treatment group, no cancer-related events occurred, but slight stomach bloating was noted in two (0.5%) participants at one or three months after enrollment. The symptom of stomach bloating was relieved after extending the time interval between taking food and medicines.

## 4. Discussion

In this study, CHMs plus conventional treatment led to a more favorable outcome for patients with ACS after successful PCI compared to conventional treatment alone. The benefits included a reduction in the incidence of the primary endpoint and secondary endpoint, as well as incidence of ischemia-driven revascularization in components of primary endpoint and readmission for ACS in components of secondary endpoint. The safety of CHMs plus conventional treatment was also confirmed in the study.

We had searched the MEDLINE (1966 to 2012), OVID (1946 to 2012), and Cochrane libraries (last search done on May 15, 2012) using the terms “Chinese herbal medicine,” “percutaneous coronary intervention,” and “coronary artery disease” to identify all randomized controlled clinical trials that had compared the efficacy of CHMs plus conventional treatment versus conventional treatment alone on cardiovascular events for CAD after PCI. Four trials met the selection criteria [15–18], but none of these addressed patients with a full spectrum of ACS after PCI. Thus, to our knowledge, our trial is the first randomized, controlled study in mainland China to assess the efficacy of CHMs plus conventional treatment versus conventional treatment alone on ACS after PCI, as evaluated by cardiovascular events. Of the four trials that met selection criteria, three [15, 17, 18] demonstrated an association between CHMs pharmacologically similar in effect to Fufang *Chuanxiong* Capsule and reduction of restenosis in post-PCI patients. The benefits of CHMs in restenosis lend support to our finding of a reduction in ischemia-driven revascularizations in the treatment group.

This study did not demonstrate a significant impact of CHMs on mortality or recurrent MI, a result that might be ascribed to the relatively small sample size of our trial. The reduction in the incidence of the composite primary endpoint in the treatment group was largely attributed to the benefits of CHMs plus conventional treatment in reducing ischemia-driven revascularization.

Our results also demonstrated a significant reduction in the secondary endpoint. The reduced incidence of the composite secondary endpoint was largely derived from a significant decrease in readmission for ACS in particular. However, we cannot exclude the possibility that CHMs might also provide benefits regarding stroke or congestive HF, because previous experimental studies have demonstrated that the active ingredients contained in Xinyue Capsule and Fufang *Chuanxiong* Capsule exert cardiovascular benefits in myocardial ischemia, myocardial hypertrophy, myocardial remodeling, HF, and thrombosis [19–21]. In recent randomized controlled trials, the benefits of CHMs on myocardial perfusion, infarcted area, and ventricular wall movement were demonstrated in patients with ST-segment elevation MI after PCI [22]. Given that no previous study has investigated the effects of CHMs on cardiovascular events in ACS patients after PCI, our trial provides the first evidence that Xinyue Capsule and Fufang *Chuanxiong* Capsule in combination with conventional treatment may further improve clinical outcomes in ACS patients after PCI by reducing cardiovascular events.

In consideration of the potential pharmacological interplay between CHMs and antiplatelet agents, the major side effect of Xinyue Capsule and Fufang *Chuanxiong* Capsule in combination with conventional treatment in this study was predicted to be a possible increase in hemorrhagic events. Because no major bleeding events occurred in either of the two groups, our results suggest that CHMs plus conventional treatment with antiplatelet agents did not increase the risk of major bleeding events in patients with ACS after PCI.

Data from a previous meta-analysis [23] indicated a statistically increased risk of noncardiac mortality (cancer, stroke, or infectious diseases, etc.) in CAD patients treated with sirolimus-eluting stents (SES) versus bare metal stents, 40% of which was cancer-related death, implicating an association between the use of SES and an increase in cancer-related mortality. Our study found that four subjects were afflicted with cancer in the control group, and all had an implanted SES. No patients in the treatment group, however, suffered from cancer. The association between cancer-related events and the use of SES or CHMs require further investigation owing to the small number of cancer-related events observed in the present study and the lack of statistical evidence provided in previous reports.

Some limitations in this study should be noted. First, our study was not a blind, placebo-controlled study. To reduce biases from observation, data collection, and efficacy evaluation, all data collectors, outcome adjudicators, data handlers, and data analysts involved in this study were not knowledgeable of the study group assignment. Second, the number of enrolled participants was relatively small and the follow-up period was only one-year. As a result, the study may not have sufficient power to detect a statistically significant difference in each endpoint between the two groups. Finally, our trial was only conducted in mainland China and all participants were Chinese; thus, our findings may not be applicable to patients from different races or other countries. 

However, our findings shed light on the benefits and safety of CHMs plus conventional treatment for ACS patients after PCI, thereby offering potential implications for clinical practice. As more evidence related to the benefits and safety of CHMs emerges from large-scale and long-term trials, CHMs may serve as an adjunctive therapy to conventional treatment for ACS after PCI in the future.

In conclusion, this study demonstrated that CHMs in combination with conventional treatment further reduced cardiovascular events in patients with ACS after PCI without an increased risk of major bleeding.

## Figures and Tables

**Table 1:** Baseline characteristics of participants.

Characteristic	Treatment group	Control group
	(*n* = 404)	(*n* = 404)
Demographics		
Male, *n* (%)	322 (79.7)	281 (69.6)
Age, median (interquartile ranges)	60 (53, 67.75)	61 (53, 68)
Final diagnosis^‡^, *n* (%)		
NSTE-ACS	287 (71.0)	296 (73.3)
STE-ACS	117 (29.0)	108 (26.7)
Number of diseased vessels, *n* (%)		
One	104 (25.7)	110 (27.2)
Two	131 (32.4)	115 (28.5)
Three	169 (41.8)	179 (44.3)
Target vessels^§^, *n* (%)		
LAD	322 (79.7)	319 (79.0)
LCX	226 (55.9)	209 (51.7)
RCA	231 (57.2)	234 (57.9)
LM	30 (7.4)	36 (8.9)
Risk factors, *n* (%)		
Hypertension	247 (61.1)	262 (64.9)
Diabetes mellitus	111 (27.5)	123 (30.4)
Hyperlipidemia	163 (40.3)	159 (39.4)
Smoking history	234 (57.9)	225 (55.7)
Family history of CAD	103 (25.5)	95 (23.5)
BMI** mean (SD)	25.31 (3.01)	25.60 (2.88)
Medication, *n* (%)		
Beta-blocker	157 (38.9)	160 (39.6)
ACEI	125 (30.9)	123 (30.4)
ARB	74 (18.3)	80 (19.8)
CCB	96 (23.8)	102 (25.2)
Statin	195 (48.3)	192 (47.5)

^‡^NSTE-ACS: non-ST-segment elevation ACS; STE-ACS: ST-segment elevation ACS.

^§^LAD: left anterior descending artery; LCX: left circumflex artery; RCA: right coronary artery; LM: left main coronary artery.

**BMI: body mass index (kg/m^2^).

**Table 2:** Clinical outcomes at 1 year^††^.

Endpoint	Treatment group	Control group	Unadjusted HR	Adjusted HR	*P* value^§§^
	(*n* = 404)^‡‡^	(*n* = 404)^‡‡^	(95% CI)	(95% CI)	
Primary endpoint	11 (2.7)	25 (6.2)	0.43 (0.21 to 0.87)	0.44 (0.21 to 0.92)	0.015
Death/MI	4 (1.0)	8 (2.0)	0.49 (0.15 to 1.64)	0.35 (0.09 to 1.33)	0.238
Revascularization	8 (2.0)	22 (5.4)	0.35 (0.16 to 0.80)	0.36 (0.16 to 0.82)	0.008
Secondary end point	14 (3.5)	35 (8.7)	0.39 (0.21 to 0.72)	0.37 (0.20 to 0.71)	0.002
Readmission for ACS	8 (2)	24 (5.9)	0.33 (0.15 to 0.72)	0.29 (0.13 to 0.65)	0.004
Stroke	3 (0.7)	6 (1.5)	0.49 (0.12 to 1.97)	0.69 (0.16 to 3.02)	0.307
Congestive HF	3 (0.7)	5 (1.2)	0.59 (0.14 to 2.48)	0.52 (0.12 to 2.36)	0.469

^††^Values are expressed as *n* (%).

^‡‡^Kaplan-Meier estimate.

^§§^ *P* value derived from log-rank test.
